# The complete chloroplast genome sequence of *Malus prattii* (Rosaceae) and its phylogenetic analysis

**DOI:** 10.1080/23802359.2019.1623732

**Published:** 2019-07-12

**Authors:** Lu Fan, Ling Qin, Jie Yan, Chuanyuan Mo, Chunxiao Rong, Ying Meng, Manrang Zhang

**Affiliations:** a College of Horticulture, Northwest A&F University, Yangling, China;; b Forestry Bureau of Luonan County, Shangluo, Shaanxi, China

**Keywords:** *Malus prattii*, chloroplast genome, Rosaceae, phylogenetic analysis

## Abstract

*Malus prattii* (Rosaceae) is an endemic species to China. Here, the complete chloroplast genome of *M. prattii* was obtained by using Illumina pair-end sequencing. The *M. prattii* complete chloroplast genome was 160,239 bp in length, containing a large single-copy region (LSC) of 88,355 bp and a small single-copy region (SSC) of 19,176 bp, which were separated by a pair of inverted repeat regions (IRs) of 26,354 bp. The chloroplast genome of *Malus prattii* contained 112 unique functional genes, including 78 protein-coding genes, 30 tRNA genes, and 4 rRNA genes. The overall C + G content of the whole genome was 36.5%. A neighbour-joining phylogenetic analysis suggested a close relationship between *M. prattii* and *M. micromalus*.

*Malus prattii* is a deciduous tree, grows to 10 m, belongs to a genus of plants in the family Rosaceae. It is endemic to China and is mainly distributed in western provinces, which occurs in mixed forests on slopes at elevations from 1400 m to 3500 m (Editorial Committee of Flora of China, Chinese Academy of Sciences 1974). *Malus prattii* is very popular among people for its charming flower. This plant have stronger cold-resistant capacity (Luby et al. 1998) and disease resistance (Abe et al. 2007), which have breeding potential especially for rootstocks and ornamentals. In this study, we report and characterize the complete chloroplast of *M. prattii* based on Illumina paired-end sequencing data, which are a resource for future studies on the taxonomy and utilization of Rosaceae. The annotated genomic sequences have been submitted to GenBank under accession number MH929090 for *M. prattii*, respectively.

The voucher specimen (FL-05-13) of *M. prattii* has been deposited in the Institute of College of Horticulture, Northwest A&F University, Yangling, China. Total genomic DNA was isolated from fresh leaves of a single individual (Yangling, Shaanxi, China; 34°30′N, 108°04′E) of *M. prattii* using the DNeasy Plant Mini Kit (Qiagen, Valencia, CA), and was used for the subsequent shotgun library following the manufacturer’s protocol for Novaseq6000 Sequencing System (Illumina, CA, USA). All of 37,918,958 of 150 bp raw paired-end reads were generated. The program MITObim v1.8 (Hahn et al. 2013) was employed for the assembly of chloroplast genome, with that of *Malus micromalus* (GenBank accession MF062434) as the reference. The complete cp genomes were annotated using the online program Dual Organellar GenoMe Annotator (DOGMA) (Wyman et al. 2004). In addition, we used tRNAscan-SE v2.0 (Lowe and Chan 2016) to confirm the identified transfer RNAs (tRNAs).

The complete chloroplast genome of *M. prattii* was 160,239 bp long, consisting of a pair of inverted repeat regions (IRs, 26,354 bp each), separated by a large single-copy (LSC, 88,355 bp) region and one small single-copy (SSC, 19,176 bp) region. The cpDNA of *M. prattii* contained 112 functional genes, including 78 protein-coding genes (PCG), 30 transfer RNA genes (tRNA), and 4 ribosomal RNA genes (rRNA), of which 19 were duplicated in the IRs, including 8 protein-coding genes (*rpl2, rpl23, rps7, rps12, rps19, ndhB, ycf1*, and y*cf2*), 7 tRNA genes (*trnA-UGC, trnL-CAA, trnV-GAC, trnI-CAU, trnR-ACG, trnI-GAU*, and *trnN-GUU*), and 4 rRNA genes (*rrn4.5, rrn5, rrn16*, and *rrn23*). Among the 112 unique genes, 12 protein-coding genes (*atpF, rps12, rps16, rpoC1, ycf3, clpP, rpl16, rpl2, petB, petD, ndhA,* and *ndhB*) and six transfer RNA (*trnA-UGC, trnV-UAC, trnK-UUU, trnG-UCC, trnL-UAA,* and *trnI-GAU*) contained one or two introns. The nucleotide composition is asymmetric (31.3% A, 18.6% C, 17.9% G, 32.1% T) with an overall C + G content of 36.5%. The GC content of cp genome in the LSC (large single copy), SSC (small single copy), IRA (inverted repeat A region), and IRB (inverted repeat B region) is 34.2%%, 30.4%, 42.7%, and 42.7%, respectively. The whole complete chloroplast genome sequence data of all 15 species were used to construct the phylogeny tree. A neighbor-joining (NJ) phylogenetic tree result showed that *M. prattii* is closely related to *M. micromalus* (Figure 1).

**Figure 1.**
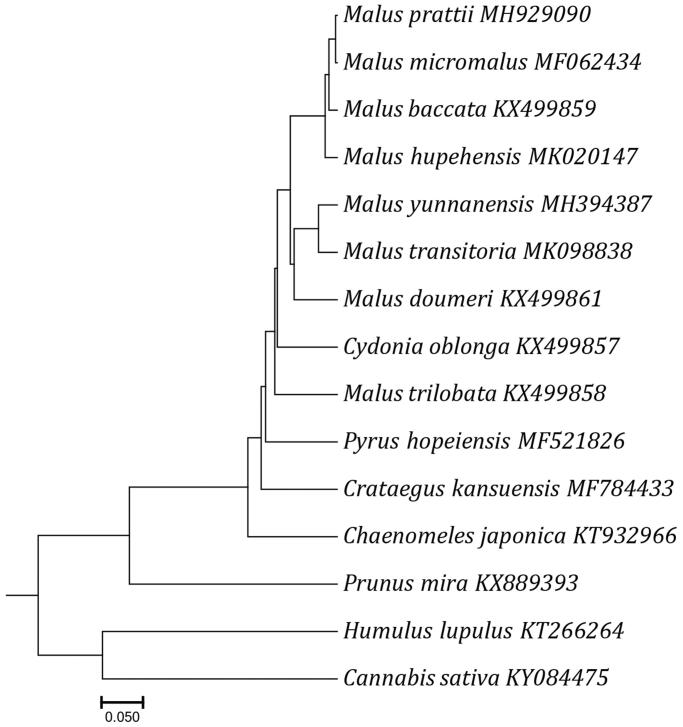
Neighbor-joining (NJ) phylogenetic tree reconstruction including 15 species based on all chloroplast genomes. *Humulus lupulus* and *Cannabis sativa* (Moraceae) were used as the outgroup.
